# Implications of drug-induced phenotypical resistance: Is isoniazid radicalizing *M. tuberculosis*?

**DOI:** 10.3389/frabi.2022.928365

**Published:** 2022-09-09

**Authors:** RJH Hammond, Frank Kloprogge, O. Della Pasqua, Stephen H. Gillespie

**Affiliations:** ^1^Division of Infection and Global Health, School of Medicine, University of St Andrews, St Andrews, United Kingdom; ^2^Clinical Pharmacology and Therapeutics Group, School of Pharmacy, University College London, London, United Kingdom; ^3^Institute for Global Health, University College London, London, United Kingdom

**Keywords:** tuberculosis, antibiotic resistance, dormancy, phenotypic resistance, drug development

## Abstract

**Background:**

Tuberculosis treatment duration is long and does not guarantee eradication of infection. Shorter treatment regimens are a critical research objective to improve uptake and reduce the risk of relapse and bacterial resistance. The explanation for the need to continue treatment after patients are culture negative remains elusive. We have previously shown that the presence of lipid inclusions in mycobacterial cells is associated with an increase in antibiotic resistance.

**Aim:**

We investigate the bactericidal effect of isoniazid and rifampicin on the expression of lipid inclusions and characterize the degree of the associated phenotypic antibiotic resistance to a range of anti-tuberculosis agents in current use.

**Methods:**

Antibiotic killing effect for both *M. tuberculosis* and *M. komossense* were investigated by both hollow fiber bioreactor (HFS) studies and static time kill curve (STKC) experiments. Following STKC cultures were stained with resazurin, Sytox green and Nile red to establish their live/dead (resazurin positive/Sytox positive) and lipid inclusion status, respectively. In addition, *M. komossense* was studied in the hollow fiber bioreactor model (HFS) and exposed to isoniazid (H) and rifampicin (R). The MIC of current antituberculosis agents for cells from the treated hollow fiber experiments were tested.

**Results:**

Antibiotic killing was similar for both species. For *M. komossense*; isoniazid was ineffective at the established MIC (1 mg/L) in the hollow fiber bioreactor but rifampicin reduced the viable count rapidly at MIC (0.4 mg/L). When the two drugs were combined at their respective MICs the killing effect was significant and greater than separately. Cells exposed to isoniazid (1x and 9x MIC) for 168 h showed considerable numbers of recoverable viable cells when compared with a combination of 1x MIC R & H where there were no viable cells detectable. For both drugs the number of lipid body positive cells increased over time and this effect was most pronounced for isoniazid and was associated with phenotypic resistance to multiple anti-tuberculosis drugs.

**Conclusion:**

Our results showed that isoniazid is a potent stimulator of lipid body accumulation, culture persistence, and phenotypic resistance to multiple anti-tuberculosis drugs. These findings emphasize the importance of understanding mechanisms of drug-drug interactions and phenotypic resistance in regimen building.

## Introduction

The need for multiple drug treatment for tuberculosis, the infectious disease responsible for the most deaths, is to prevent relapse and the development of resistance. The late relapses found in the seminal drug trial with streptomycin was associated with what we now know was genotypic resistance (Streptomycin in Pulmonary Tuberculosis, 1948; Fox et al., 1954). Para-amino salicylic acid, initially and isoniazid (H) later, were combined to produce a 12-month course of treatment with an acceptable relapse rate (Fox, 1981). The inclusion of rifampicin (R) and later pyrazinamide (Z) resulted in the current 6-month regimen where the emergence of resistance is very rare among patients who follow the regimen fully (Fox et al., 1999).

Recent attempts to shorten the duration of therapy from 6 to 4 months through the addition of fluoroquinolones alone were not successful (Gillespie et al., 2014; Merle et al., 2014). A successful 4-month regimen that includes moxifloxacin and rifapentine has been reported. (Dorman et al., 2021). Analysis of sequential treatment response data from the REMoxTB trial suggests that some patients go on to relapse at the end of the follow up period even though they clear bacteria from their sputum rapidly (Prideaux et al., 2015). In addition to the lack of quantitative evaluation of the pharmacokinetic-pharmacodynamic relationships and contribution of moxifloxacin to the backbone therapy (Muliaditan and Pasqua, 2021), it can be speculated that these results may be caused by an occult population where there is failure of penetration to the site of infection, or because the bacteria exhibit phenotypic resistance (Hammond et al., 2015; Muliaditan and Pasqua, 2021). A recently published mathematical model of tuberculosis confirms that both characteristics may contribute to the relatively high relapse rate in the disease (Bowness et al., 2018) and this is now supported by experimental evidence (Prideaux et al., 2015).

It has long been known that mycobacteria develop lipid inclusions (Garton et al., 2002) rich in tri-acyl glyceride (Garton et al., 2008) in response to multiple physiological stresses, for example, anoxia (Wayne and Hayes, 1996), (see Lipworth et al., for a detailed review of this subject Lipworth et al., 2016). Lipid accumulation is associated with a metabolic slowdown and, from the therapeutic point of view, phenotypic resistance to the antibiotics used in tuberculosis treatment (Keren et al., 2011; Hammond et al., 2015; Aguilar-Ayala et al., 2017).

It is also likely that such phenotypical changes correlate with differences in growth dynamics of *M. tuberculosis* subpopulations that occur during an infection in humans (Iona et al., 2016; Muliaditan and Pasqua, 2021). Associated with the formation of lipid bodies six major “types” of dormancy are described with a range of features including a reversible state of metabolic inactivity to a state of near-quiescence with or without altered drug resistance profiles (Mukamolova et al., 2010; Lipworth et al., 2016; Baron et al., 2017). These forms have been demonstrated *in vivo* and *ex vivo* studies (Mukamolova et al., 2010; Baron et al., 2017).

To date, the implications of phenotypic resistance in the evaluation of alternative drug regimens, dose selection and treatment duration have not been pursued previously in a systematic manner due to technical and experimental limitations. Some of these limitations may be overcome by the availability of hollow fiber systems and novel staining techniques (Gumbo et al., 2015; Hammond et al., 2015). It is also unclear whether phenotypic resistance is concentration-dependent and specific to *M. tuberculosis* or whether the changes in phenotype caused by one drug can affect the antibacterial activity of other drugs in a combination regimen. In this report, we investigate the role of two critical drugs (isoniazid and rifampicin) as inducers of phenotypic resistance associated with the appearance of lipid body positive mycobacterial cells using static and dynamic protocols that allow us to compare the implications of steady-state and time-varying drug exposure using both *M. tuberculosis* and a model organism *M. komossense*. In addition, we explore the effects of anti-biotic induced lipid inclusions on the overall bactericidal activity of compounds used for tuberculosis treatment.

## Methods

An overview of the experimental procedures used for the purposes of this investigation is illustrated in in the flow diagram in Supplementary Figure 1.

### Bacteria

Isolates of *M. komossense* (ATCC 33013), a rapid growing hazard group 1 organism and *M. tuberculosis* (H37Rv, NCTC 7416) were incubated in sealed 50 ml tubes (Falcon, Corning, USA) with Middlebrook 7H9 (Fluka) with 0.05% Tween (Sigma Aldrich) at 30°C for 3–5 days or at 37°C for 28 days until confluent or at ≥0.1 OD_600_. Viable counts were determined by a modified Miles and Misra method as described previously (Billington et al., 1999). A portion of the Mtb culture was heat-killed at 80°C for 20 min and OD_600_ measured.

### Antibiotics

Antibiotics solutions were prepared from lyophilised powder (Sigma-Aldrich) as described previously (Hammond et al., 2015).

### Static time kill curves

*Mycobacterium komossense* or *Mycobacterium tuberculosis* (Mtb) was inoculated into Middlebrook 7H9 media (Oxoid) with 0.5% Tween 80 (Sigma) and ADC supplement (Oxoid) from glycerol stock stored at −80°C. Cultures were incubated static at 30°C for 4 days (37°C, 14 days Mtb) or until turbidity reached ≥0.1 OD_600_. A 20 μl aliquot of the relevant antibiotic alone or in combination was added to each well of a 96-well plate at 10x final concentration. One hundred microliter of culture was then aliquoted into each well and the volume made up to 200 μl with Middlebrook 7H9 medium. The plates were sealed and incubated at 30°C for 24 h for *M. komossense* or 37°C for 5–7 days for *M. tuberculosis*. The optical density of the cultures was assessed after the appropriate incubation periods. A 10 μl aliquot of culture was removed and stained as noted (see Section *Methods–Staining*). A separate preparation was stained similarly and analyzed in a flow cytometer for lipid body fluorescence, all steps were repeated every day for 7 days for *M. komossense* and every day for 28 days for *M. tuberculosis*. All measurements were conducted in triplicate.

### Hollow fiber system-bacteria and culture

Hollow fiber cartridges (C3008 cellulosic cartridges, FiberCell, New Market, Maryland, USA) as described previously (Kloprogge et al., 2019). The hollow fiber model was prepared per the manufacturer's instructions and attached aseptically. The arrangement is shown in Supplementary Figure 2. Bacteria were introduced into the cartridge and allowed to settle for 1 h. Drugs were delivered at infusion rates, which maintained drug levels comparable to steady state conditions in patients receiving clinical doses of rifampicin and/or isoniazid. This was maintained automatically; once drug infusion was finished the system automatically restarted; pumps supplying both drug-free media and eliminating from the reservoir functioning at the required rate until the next drug infusion. Bacterial sampling took place throughout the 7-day or 28-day period. To sample the bacteria from the cartridge, the flow pump (FiberCell Duet 2) was switched off temporarily, 5 ml of medium were removed as described by the manufacturer (FiberCell). The filled syringe was removed from the assembly, the Luer lock was sprayed with ethanol and a new sterile syringe was added to the vacated Luer lock. The flow pump was restarted and continued until the next sampling interval.

### Staining

Mycobacterial cells were stained with (0.25 μg/ml) Nile Red (Sigma Aldrich, UK) at room temperature with constant agitation for 20 min. The samples were washed with 100% ethanol and again with PBS and an aliquot (10 μl) was then spotted on to a clean glass slide and heat fixed. Bacterial preparations were viewed by fluorescence microscopy at 100X (Leica DM5500) (excitation; 480/40, 540/40nm. Emission 527/30, 645/75nm), as described previously.

Assessment of the viability was made using Sytox green^®^ (Sigma Aldrich, UK) and resazurin sodium salt (Alamar blue) (Sigma Aldrich, UK). Cultures under investigation were stained with resazurin at 0.01% solution in the dark for 5 h for *M. komossense* or overnight (16 h) for *M. tuberculosis* at the appropriate temperature. In the last hour of the incubation period for both species Sytox green at 20 μM was introduced and cultures were placed back into the incubator in the dark. Analysis was by flow cytometry.

### Flow cytometry

Flow cytometry was carried out on a Millipore Guava easyCyte™ HT as previously reported (Hammond et al., 2015). Cells were stained with, Sytox green, resazurin or Nile Red as above and loaded into a flat-bottomed 96 well plate (Nunc, Thermo-Fisher Denmark). The flow cytometer scanned the preparations at 488 nm (blue light) and collected signal at 525/30 nm and 690/50 nm. Results were obtained *via* gated pair-wise analysis based upon previous work using archival parameters.

### Phenotypic susceptibility testing

The ability of organisms to grow at different antibiotic concentrations (x1, x4, x16 MIC) was tested using a modified microplate dilution technique for isoniazid, rifampicin, ethambutol, pretomanid and bedaquiline at 30°C for 24 h for *M. komossense* or 37°C for 5–7 days for *M. tuberculosis*. Drugs were weighed in their powered state and diluted in sterile deionised water to a stock concentration of 10 mg/ml (10 g/L). Drug dilutions were made to 10x final concentration in a series of three (1x, 4x & 16x MIC) 1.5 ml micro-centrifuge tubes by dilution with sterile, deionised water. A fourth tube of sterile, deionised water was used as a drug blank in a growth control well. 20 μl of these solutions were then added to a sterile 96-well plate in triplicate. One hundred sixty microliter sterile Middlebrook 7h9 was added to the plate in the same wells as the drugs. Finally, the samples of *M. komossense* or *M. tuberculosis* at each time point were added to the 96-well plate at a volume of 20 μl diluting the culture 10x and making the final volume in each well 200 μl. This was repeated for each of the 5 drugs used. 96-well plate was incubated for 3 or 7 days (*M. komossense* and *M. tuberculosis*, respectively) and read in a 96-well plate reader, data was collected as OD_600._ Cultures were defined as susceptible or resistant based on EUCAST definition of 50% inhibition vs. control growth^1^ Samples were stained with Nile Red as described above.

## Results

### Comparison between *M. komossense* and *M. tuberculosis* as a tool anti-tuberculosis drug evaluation

**Figure 1:**
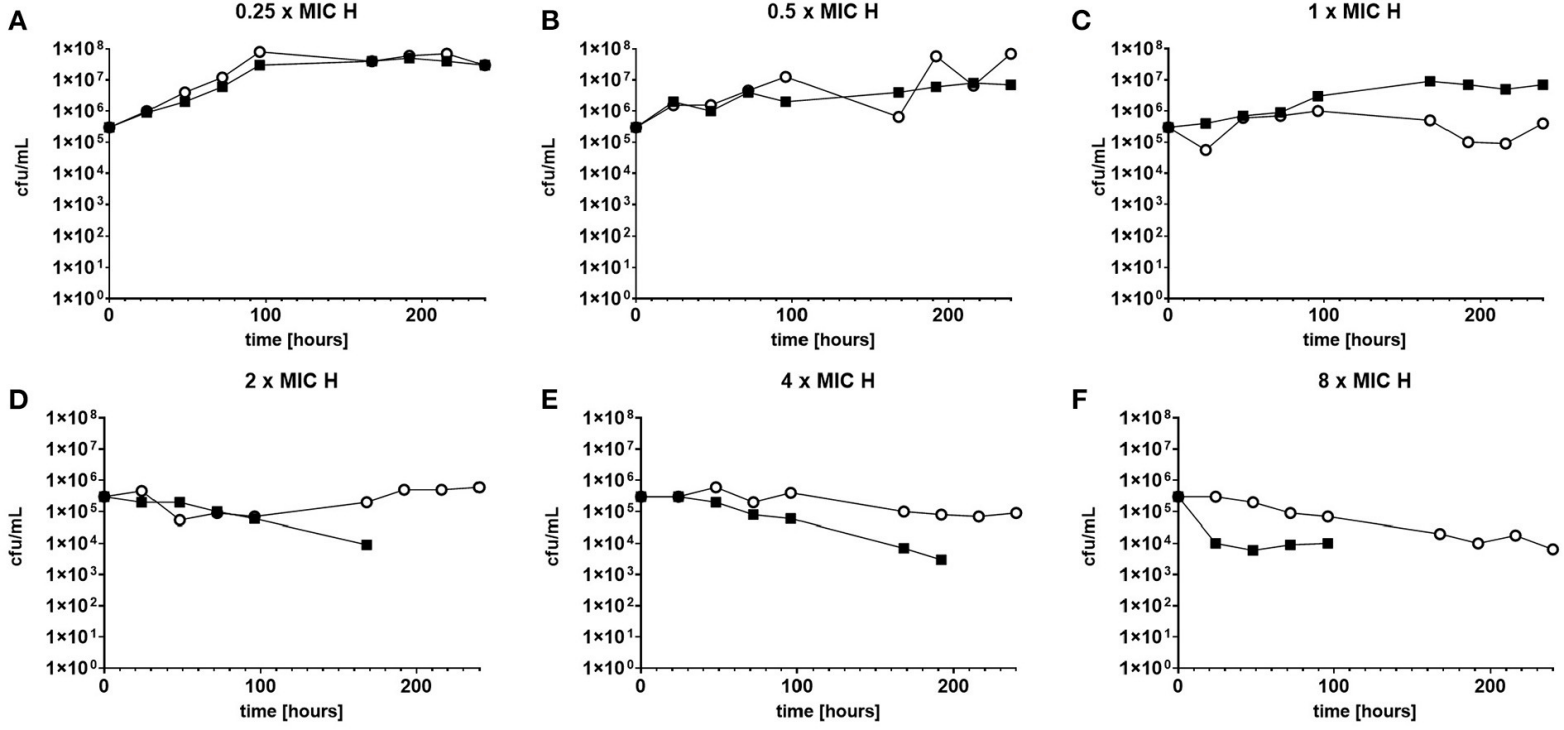
**(A–F)** Static time kill experiments represent the change in the viable count of *M. tuberculosis* (closed squares) and *M. komossense* (open circles) in response to exposure to isoniazid (H) at the displayed multiple of MIC [1 mg/L (concentrations used-0.25–8 mg/L)].

**Figure 2:**
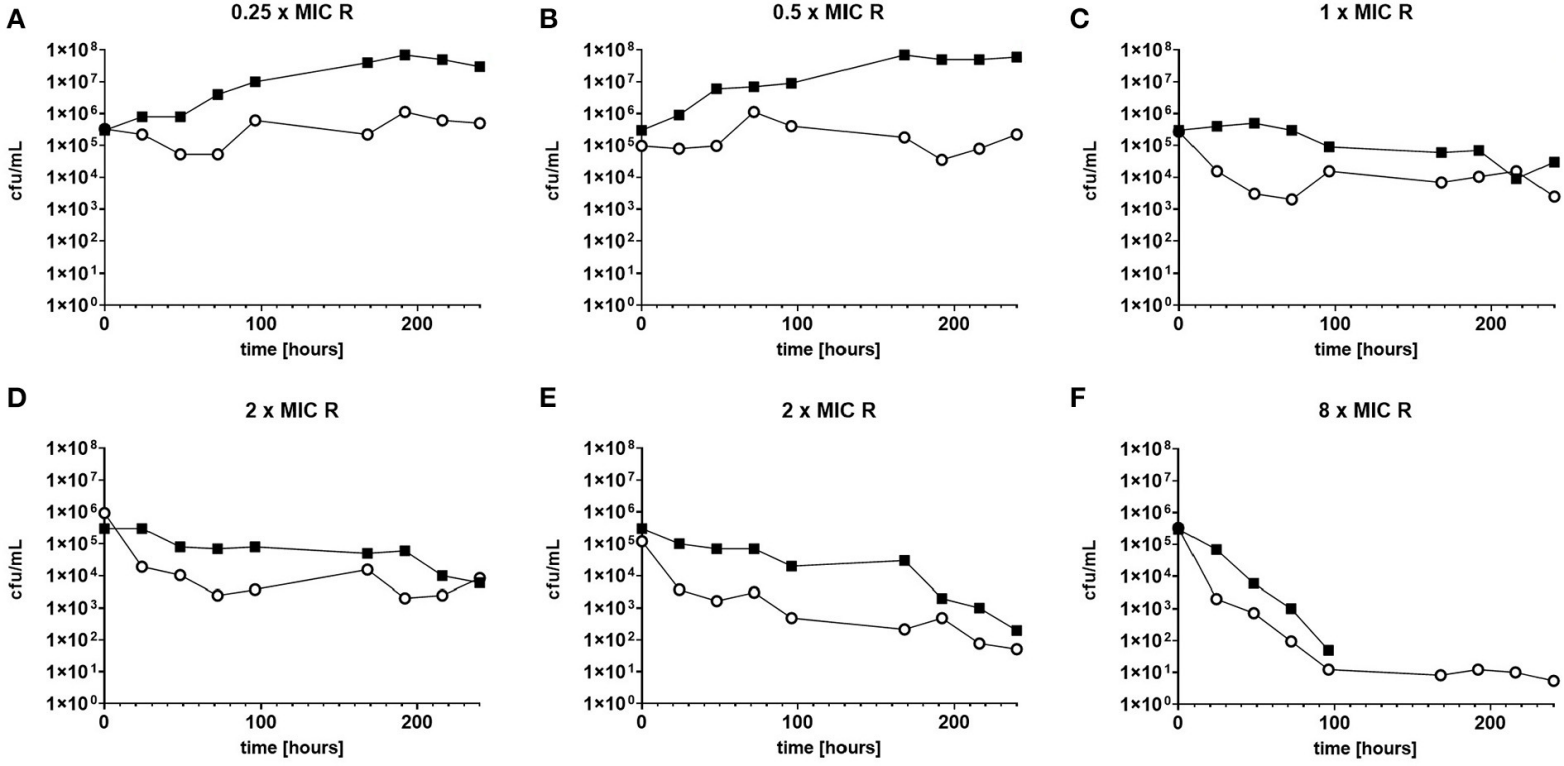
**(A–F)** Static time kill experiments represent the change in the viable count of *M. tuberculosis* (closed squares) and *M. komossense* (open circles) in response to exposure to rifampicin (R) at the displayed multiple of MIC [0.4 mg/L (concentrations used- 0.1-3.2 mg/L)].

**Figure 3:**
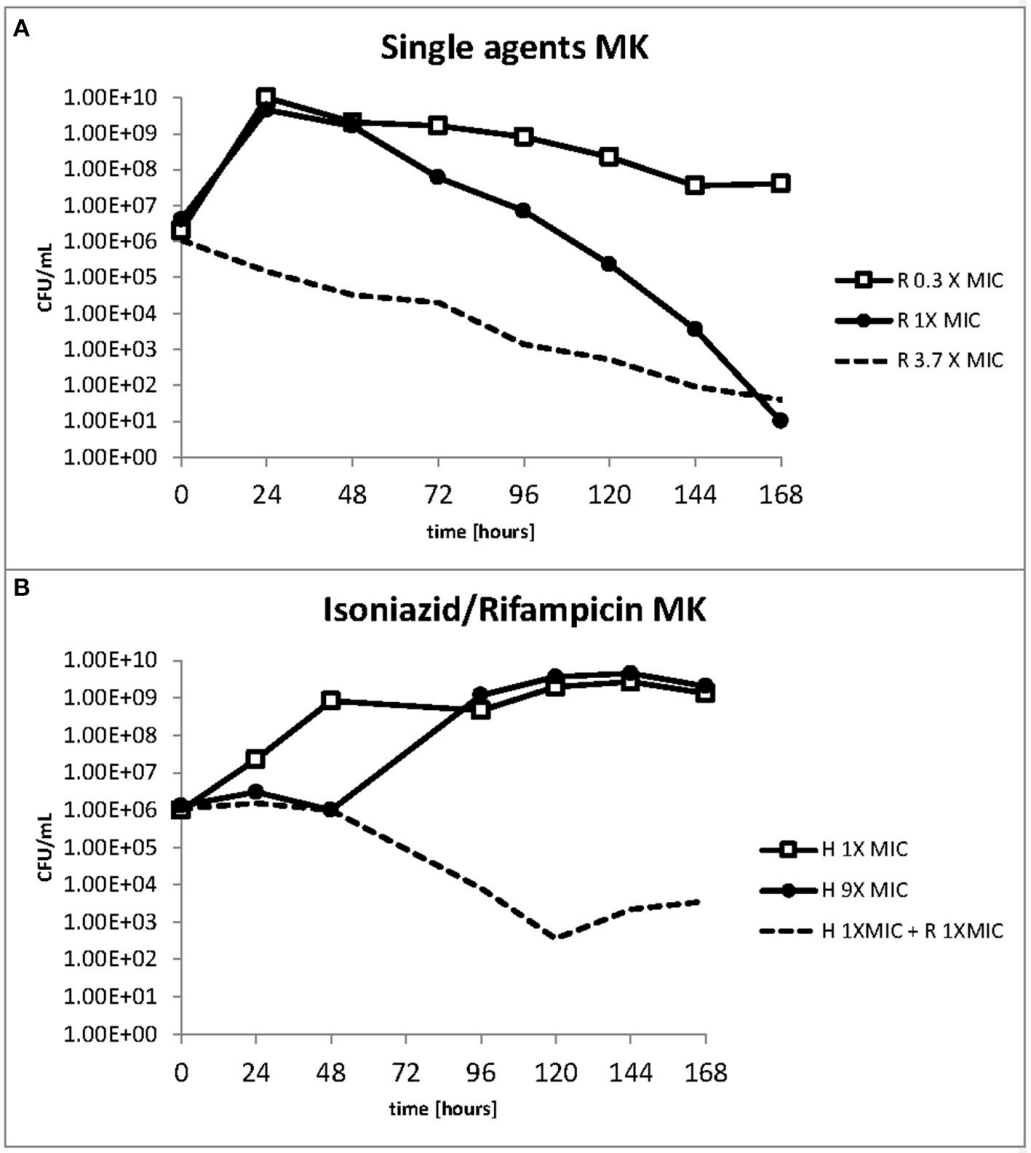
**(A,B)** Change in the viable count of *M. komossense* in the hollow fiber system in response to rifampicin alone or in combination with isoniazid at the displayed multiples of MIC. Data displayed are mean values of three technical replicates.

The response to both isoniazid and rifampicin in static time kill curves was similar for MK and Mtb (see Figures 1, 2). These data demonstrate that for both species rifampicin, at doses at or greater than the MIC, result in a steady fall in the viable count (Figures 2D–F) and there is complete sterilization of the Mtb culture with the use of rifampicin at 8 × MIC in 96 h (Figure 2F). By contrast, when isoniazid (2 × and 4 × MIC) is used, a slow reduction in viable count is found and it is only at x8 MIC that reduction in bacterial numbers starts to resemble the activity of rifampicin. This pattern is similar for both species although more pronounced for *M. tuberculosis*. Rifampicin at the MIC reduces the viable count slowly and isoniazid has poor activity with 9 × MIC initially producing killing that is followed by subsequent regrowth. In contrast, the combination of isoniazid and rifampicin at x1 MIC of both agents is highly effective (see Figures 3A,B).

### Drug effect on cell viability

**Figure 4:**
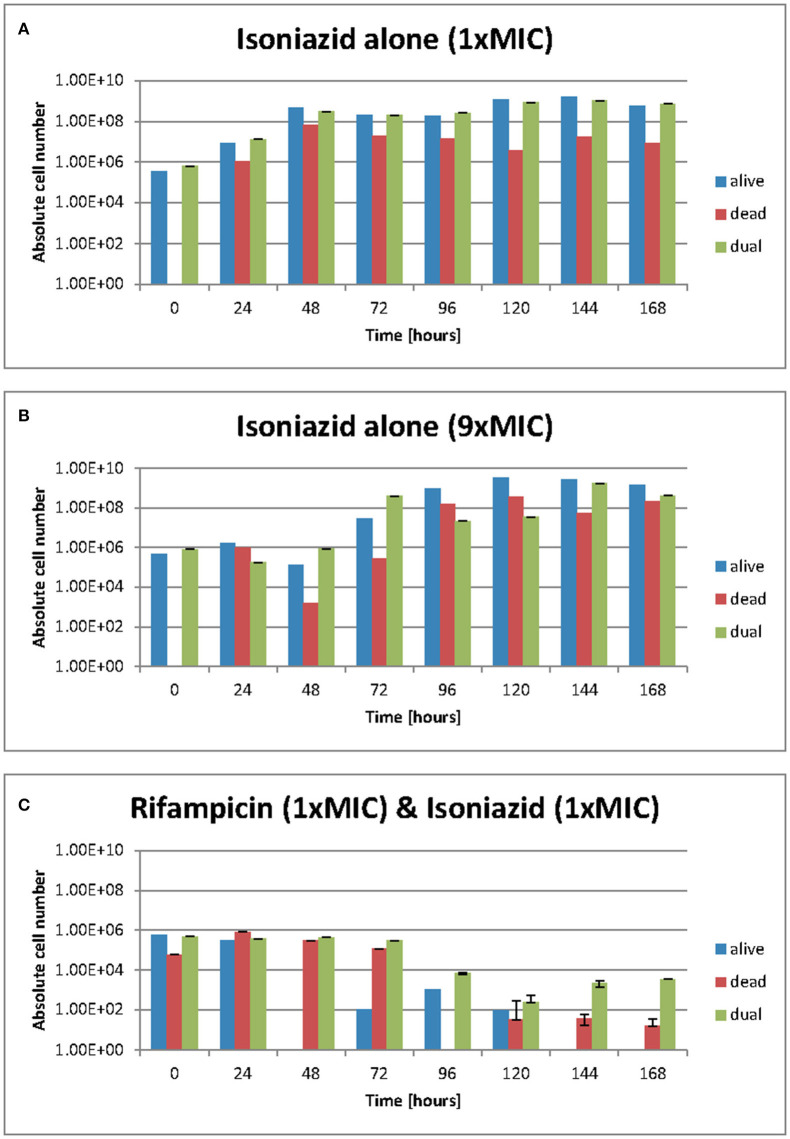
**(A–C)** Live/dead staining was achieved with Sytox green and resazurin sodium salt. Green flourescence represents a damaged cell membrane and the staining of nucleic acids. Red fluorescence is the result of the stain being reduced in the presence of an actively metobolising cell. Dual staining results from and “injured” phenotype where a cell is both respiring but has damage to its membrane such that sytox green can enter. These data were generated using a flow cytometer. Data presented as abolute cell numbers. Error bars are ± 2 S.D. of the mean of four independent experiments. The values being graphed are small at this stage of the experiment meaning across averages where *n* = 4 it can appear that there are very few or no cells in any given category, especially where the y-axis scale is set to 10^10^ ecfu/ml.

We used resazurin positive and Sytox positive bacteria to estimate the number of resazurin “live” and Sytox green “dead” to explore these observations further. At **1** × **MIC** of isoniazid (Figure 4A) the number of resazurin positive cells (blue bars) detected increased steadily and plateaued after 48 h. A similar pattern was observed at 9 × MIC of isoniazid (Figure 4B), even though a plateau occurs slightly later (at 96 h). The combination of rifampicin and isoniazid that we have already shown is killing (Figure 4C) produced a very sharp decline in resazurin positive cells after 48 h until no “live” cells are found after 144 h. As anticipated, there were no Sytox green positive cells (red bars) at the beginning of both experiments with isoniazid (at 1 × and 9 × MIC) (Figures 4A,B). When exposed to the combination (isoniazid + rifampicin at 1 × MIC, Figure 4C), the number of Sytox green positive cells was steady for 72 h, after which it declined rapidly.

The total number of dual staining cells (green bars) in the experiment at 1 × and 9 × MIC (Figures 4A,B) follows a similar pattern to that of the resazurin positive cells; an increase over 48 h followed by a plateau. In the drug combination experiments (Figure 4C) the number of dual staining cells begins high and remains so for 72 h after which it declines slightly. Interestingly, the proportion of resazurin positive/Sytox positive/'Injured' (dual stained) remained the same at 168 h following treatment with isoniazid (at 1 × and 9 × MIC) (Figures 4A,B) but was markedly different for the combination (Figure 4C). At 168 h, the major populations were resazurin positive and dual stained groups with the Sytox positive group being only slightly less numerous. In the rifampicin and isoniazid combination (Figure 4C), the major population is the dual stained group with the Sytox positive group being much less numerous and the resazurin positive group missing entirely suggesting a potent killing effect- see Figures 4A–C.

Over the duration of these experiments (168 h), the relative and actual abundance of resazurin positive-stained bacteria changes significantly. When isoniazid (at 1 × MIC) was tested alone, the absolute numbers of resazurin positive staining organism rose from ~5 ×10^5^ cfu/ml at 48 h to a plataeu of ~5 ×10^8^ cfu/ml at the remaining 120 h. The number of organisms staining as Sytox positive- rose from 0 cfu/ml to ~1 ×10^6^ cfu/ml within the first 24 h and show variation within 2 log units until 168 h. The numbers of organisms staining at “dual” or “injured” follows the same patterns as the resazurin positive orgainisms at roughly the same cell densities.

### Phenotypic resistance and bacterial subpopulations

**Figure 5:**
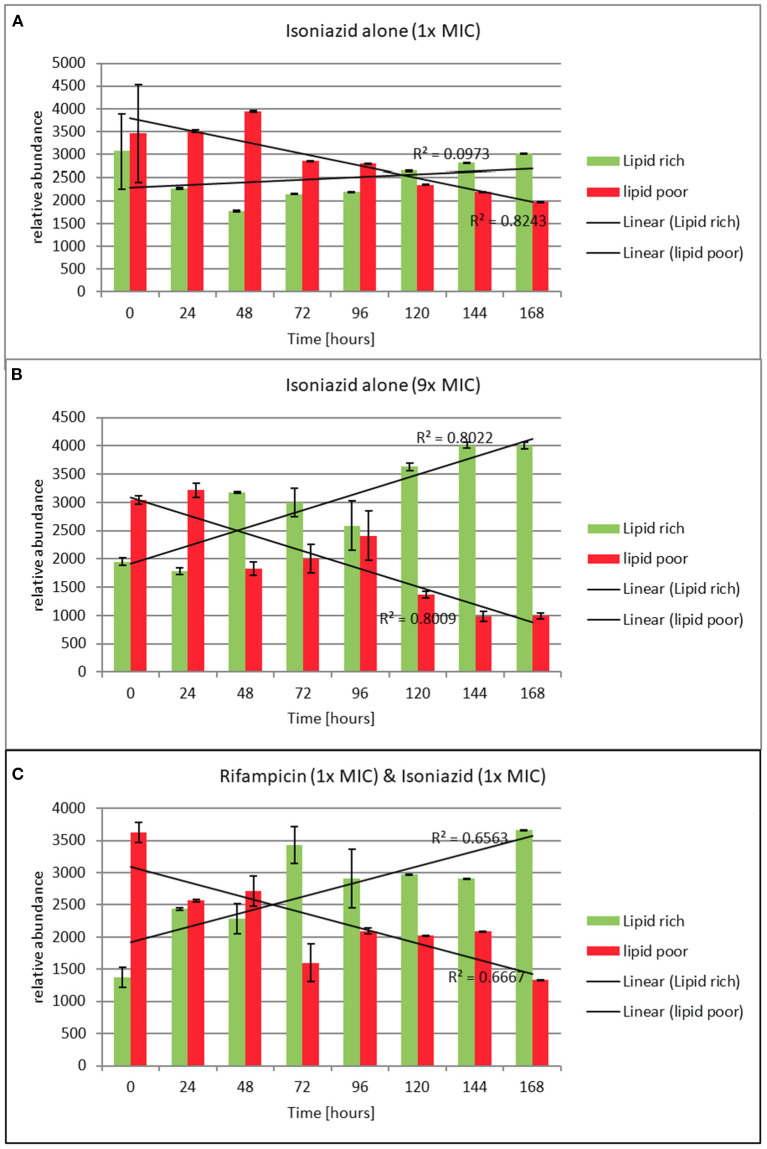
**(A–C)** Relative abundance of lipid rich (green fluorescent) and lipid poor (red fluorescent) cells in response to exposure to isoniazid alone or in combination with rifampicin at the stated multiples of MIC [MIC isoniazid = 1 mg/L, rifampicin 0.4 mg/L]. Lipid body staining was achieved with lipophillic Nile red. Green flourescence indicates the presence of one or more lipid bodies in the bacterial cells (this was confirmed *via* microscopy). Red fluorescence is the result of the stain interacting with the polar lipids in the bacterial outer envelope and can therefore be assumed to be a total cellular abundance. Where the green fluorescence exceeds the red indicates more than 1 lipid body per cell. These data were generated using a flow cytometer. Data presented is relative abundance of 5,000 events. Error bars are ± 2 SD of the mean of three independent experiments.

The effect of rifampicin and isoniazid on lipid body accumulation and phenotypic resistance varied with treatment and drug exposure. Figure 5A shows that when rifampicin is used, the concentration of bacteria does not fall below the starting inoculum when 0.3 × MIC is applied. There is killing, however, throughout the 168 h of the experiment when at 1 × and 3.7 × MIC of rifampicin are used. By contrast, at 1 × MIC growth starts immediately when isoniazid is used, whereas at 9 × MIC an initial suppressive effect is followed by bacterial growth (Figure 5B). The combination of rifampicin and isoniazid at 1 × MIC produces a killing effect of approximately three logs. It should be noted, however, that after 120 h there is a small increase in the bacterial viable count.

To understand how the antibiotics were affecting the lipid body status, we monitored the proportion of lipid poor cells (LP, total number) to lipid rich (LR, phenotypically resistant sub-population) in a static time kill curve experiment. In these experiments the proportion of lipid rich cells increased when bacteria were exposed to either of the antibiotics tested (see Figures 5A–C). When relative abundance of LR vs. LP cells is considered, we show that LR cells accumulate slowly over the course of 168 h following exposure to isoniazid at 1 × MIC. On the other hand, the increase in the proportion of LR cells is rapid when isoniazid exposure is at 9 × MIC. A similar pattern is seen with combination therapy (Figure 5C). This observation was confirmed using Nile red microscopy.

**Figure 6:**
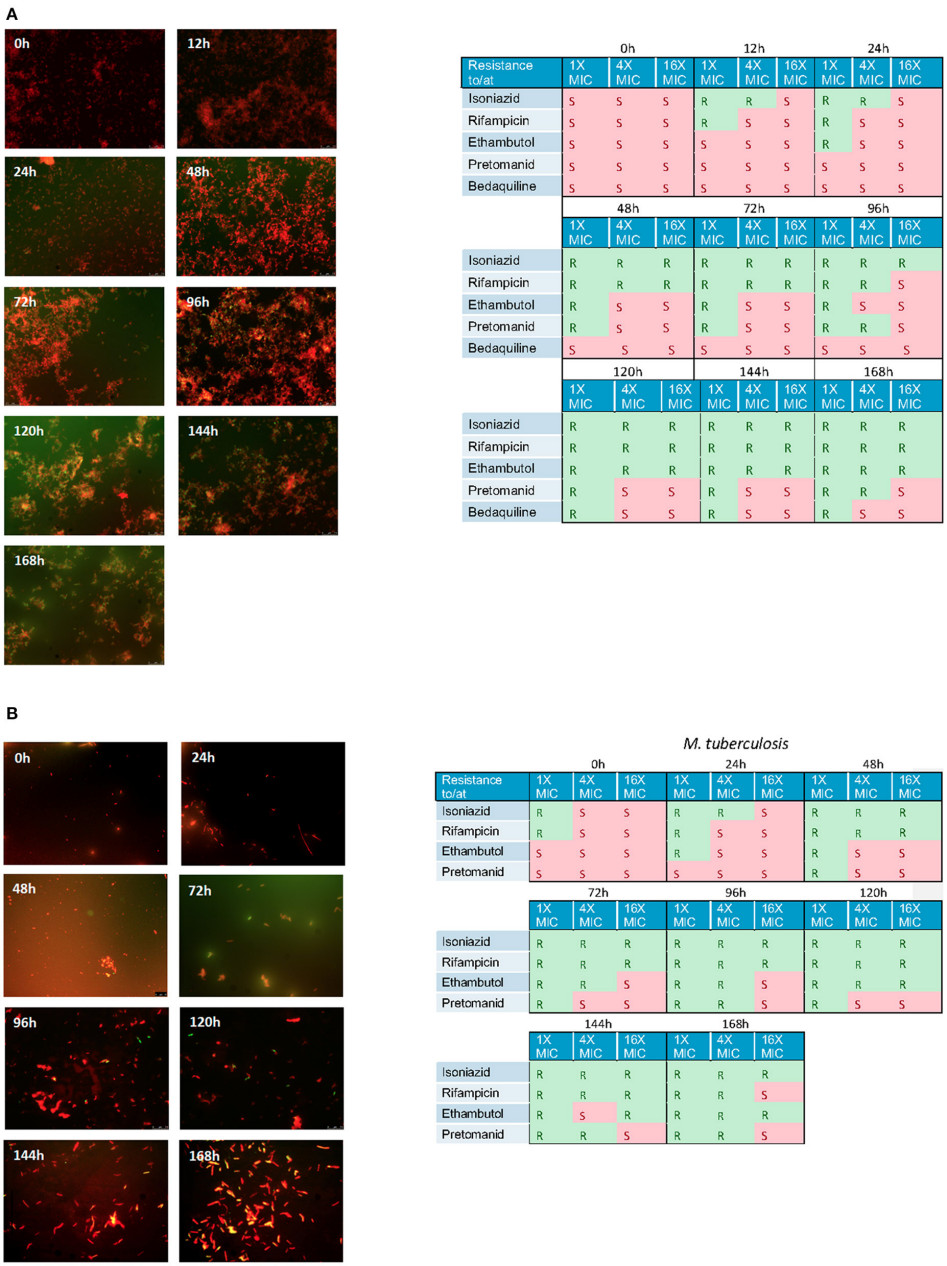
**(A)** Nile red staining of *M. komossense* of bacteria harvested from a 168 h hollow fiber experiment where bacteria were exposed to isoniazid at 1 × MIC (1 mg/L) Green flourescence indicates the presence of one or more lipid bodies in the bacterial cells. Red fluorescence is the result of the stain interacting with the polar lipids in the bacterial outer envelope. Individual samples of bacteria were defined as S= susceptibile or R= resistant based on a microbroth dilution suceptibility test conducted agaisnt isoniazid, rifampicin, ethambutol, pretomanid, and bedaquiline. **(B)** Lipid body staining of *M. tuberculosis* was achieved with lipophillic Nile red. Green flourescence indicates the presence of one or more lipid bodies in the bacterial cells. Red fluorescence is the result of the stain interacting with the polar lipids in the bacterial outer envelope. Individual samples of bacteria were defined as S = susceptibile or R = resistant based on a microbroth dilution suceptibility test conducted agaisnt isoniazid, rifampicin, ethambutol, pretomanid, and bedaquiline.

Using the hollow fiber system we were able to show a steady accumulation of lipid body positive (green) cells when cultures are incubated with isoniazid at 1 × MIC (Figures 6A,B). Simultaneously, to assess the effect these changes can have on the general antibiogram phenotype, we measured the susceptibility of both mycobacterial species to critical anti-tuberculosis drugs: isoniazid, rifampicin, ethambutol, bedaquiline and pretomanid using microbroth dilution plates as described previously. As the experiments progress, both *Mk* and *Mtb* become increasingly resistant. For both species treated with isoniazid (1 × MIC), there is a progressive loss of susceptibility to all antibiotics as the experiments advance. Resistance to isoniazid is the first to be observed, followed by rifampicin, then ethambutol, then pretomanid. This phenotypic change in susceptibility is mirrored in a progressive increase in the proportion of lipid bodies detected (see Figures 6A,B).

## Discussion

### Phenotypic resistance and drug exposure: Implications of static and dynamic protocols

Isoniazid and rifampicin are critical components of the standard TB treatment regimen, which is considered long and complex. Treatment relapse is the main reason that the 4-month continuation phase with rifampicin and isoniazid is required, but insight into the determinants of relapse is still limited, even though inadequate compliance and antibiotic resistance have been identified as important contributors (Gillespie et al., 2014). Another important shortcoming is the empirical evaluation of cure, which relies on sputum samples, without further consideration of the role of subpopulations with variable susceptibility to antibacterial effect as well as the role of certain drugs as promoters of phenotypical resistance (Hammond et al., 2015). We have shown previously that exponential cultures, such as those found in standard methodologies to determine susceptibility have only a small proportion of lipid inclusions (Hammond et al., 2015) but cells expressing lipid inclusion make up ~60% of *M. tuberculosis* cells in lung tissue (Baron et al., 2017). This means that standard susceptibility tests may not predict the effect *in vivo* effectively.

### Implications of phenotypical resistance for the overall bactericidal activity of drug combinations

Having previously identified the ability of isoniazid to stimulate lipid inclusions, a genus-wide phenomenon (Daniel et al., 2011; Barisch et al., 2015), we have taken this further alone and in combination with rifampicin which also appears to kill cells with a dormancy phenotype (Bowness et al., 2015) in both *M. tuberculosis* and the low hazard rapid growing model organism *M. kommosense*. As predicted from our earlier work (Hammond et al., 2015), exposure to isoniazid has the most profound effect and, importantly, increases the MIC to isoniazid itself as well as the other antibiotics used. Cultures are only killed effectively at isoniazid >9 × MIC. Nile Red staining of the bacteria exposed to antibiotics provides an explanation of these results. When the study mycobacteria are exposed to 1 × MIC isoniazid, the proportion of lipid poor bacteria declines and the proportion of lipid inclusion positive increases proportionally. On the other hand, when exposed to 9 × MIC isoniazid, this effect is more pronounced as the phenotypically sensitive organisms are cleared from the culture leaving only the phenotypically resistant bacteria. This creates a survivor effect: the remaining bacteria, phenotypically resistant to isoniazid, can subsequently grow out, mimicking an *in vitro* relapse. This means that antibiotics themselves, can result in phenotypic resistance not only to the stimulating agent but also to other anti-tuberculosis agent. As these cell types have been demonstrated repeatedly in multiple human samples (Mukamolova et al., 2010) as well as in animal models (Baron et al., 2017, 2020) our results may explain the difficulties of eradicating *M. tuberculosis* in patients (Gillespie et al., 2014). These data appear to mirror the phenomenon previously reported by Jindani and colleagues who demonstrated the rapid fall in bacterial load in patients when exposed to isoniazid only (Jindani et al., 1980). This rapid decline was followed by a slower decline and was named “early bactericidal activity (EBA)”. Yet no other drugs appeared to have the same impact (Gillespie et al., 2005; Diacon et al., 2012). With results presented here, an alternative explanation for this clinical EBA effect can be advanced. Isoniazid rapidly kills the lipid poor bacteria causing a large fall in the number of viable mycobacteria but selects for cells with lipid inclusions that are phenotypically resistant. Ongoing treatment with isoniazid has much less effect since the effective MIC of the surviving population is much higher (Hammond et al., 2015). Sloan and colleagues demonstrated that patient with a higher percentage of lipid inclusions cells 3–4 weeks into therapy were more likely to have a poor outcome (Sloan et al., 2015). We have shown the relationship between lipid inclusions and a large increase in the MIC to the main drugs used in tuberculosis chemotherapy in a previous publication (Hammond et al., 2015). Whilst the current investigation provides support for drug regimens with a combination of 1 × MIC rifampicin and 1 × MIC isoniazid to overcome the challenge of induced phenotypic resistance, maintaining such a ratio between the two drugs may not be feasible *in vivo*, as pharmacokinetic processes lead to significant variation in drug levels both systemically and at tissue level. The importance of higher rifamycin dosing has been noted in the recently reported successful 4-month regimen including higher-dose rifapentine and moxifloxacin (Dorman et al., 2021).

It is crucial, therefore, to consider a different paradigm for the identification of suitable companion drugs and dose rationale for combination regimens that accounts for the presence of bacterial subpopulations, as demonstrated here by the changes in lipid inclusions and phenotypic resistance observed during treatment. Many emerging pharmacokinetic-pharmacodynamic models base their predictions on data assuming the contribution of different bacterial subpopulations (Baron et al., 2017). Such models provide quantitative estimates of bacterial clearance and potency for each subpopulation. We show that *M. kommosense* and *M. tuberculosis* respond similarly. This provides an experimental paradigm that would allow this phenomenon and with a range of antibiotic combinations to be investigated rapidly.

### Limitations

As indicated above, *in vitro* observations may not be fully reproduced *in vivo*. We have, however previously shown that approximately one third of *M. tuberculosis* cells in the lung contain lipid inclusions emphasizing the importance of understanding such effects (Baron et al., 2017). Although we use a dynamic hollow fiber system further work is required to understand the relationships between dosage and the natural state of *M. tuberculosis* cells. Future studies will need to characterize the effect of varying exposure and treatment duration on phenotypic resistance.

## Conclusions

Improving anti-tuberculosis chemotherapy is an important research goal with many international drug development consortia attempting to make progress. The current drug development pathway is still based upon conventional markers of minimum inhibitory concentration derived microbroth dilution or MGIT, which do not distinguish bacterial sub-populations and may consequently yield inaccurate estimates bactericidal effect of established and novel compounds (Baron et al., 2017). Our data suggest that studying drug-drug interactions and their capacity to induce lipid bodies might be important in improving the choice of companion drugs. This could be accelerated by use of our *M. komossense* model system described here, could accelerate that.

## Data availability statement

The raw data supporting the conclusions of this article will be made available by the authors, without undue reservation.

## Author contributions

Laboratory work was performed by RH. All authors planned the research and were involved in the writing and review of the manuscript.

## Funding

This study was funded by a grant from the British Society for Antimicrobial Chemotherapy (GA2015-172R).

## Conflict of interest

Author OP is Senior Director Clinical Pharmacology at GlaxoSmithKline. The remaining authors declare that the research was conducted in the absence of any commercial or financial relationships that could be construed as a potential conflict of interest.

## Publisher's note

All claims expressed in this article are solely those of the authors and do not necessarily represent those of their affiliated organizations, or those of the publisher, the editors and the reviewers. Any product that may be evaluated in this article, or claim that may be made by its manufacturer, is not guaranteed or endorsed by the publisher.

## Footnotes

^1^ https://www.eucast.org/ast_of_bacteria/
