# Supplementary figures and images for: Implications of drug-induced phenotypical resistance: Is isoniazid radicalizing M. tuberculosis?
Source: Front Antibiot. 2022 Sep 9;1:928365. doi: 10.3389/frabi.2022.928365 (PMC11732046; doi:10.3389/frabi.2022.928365)

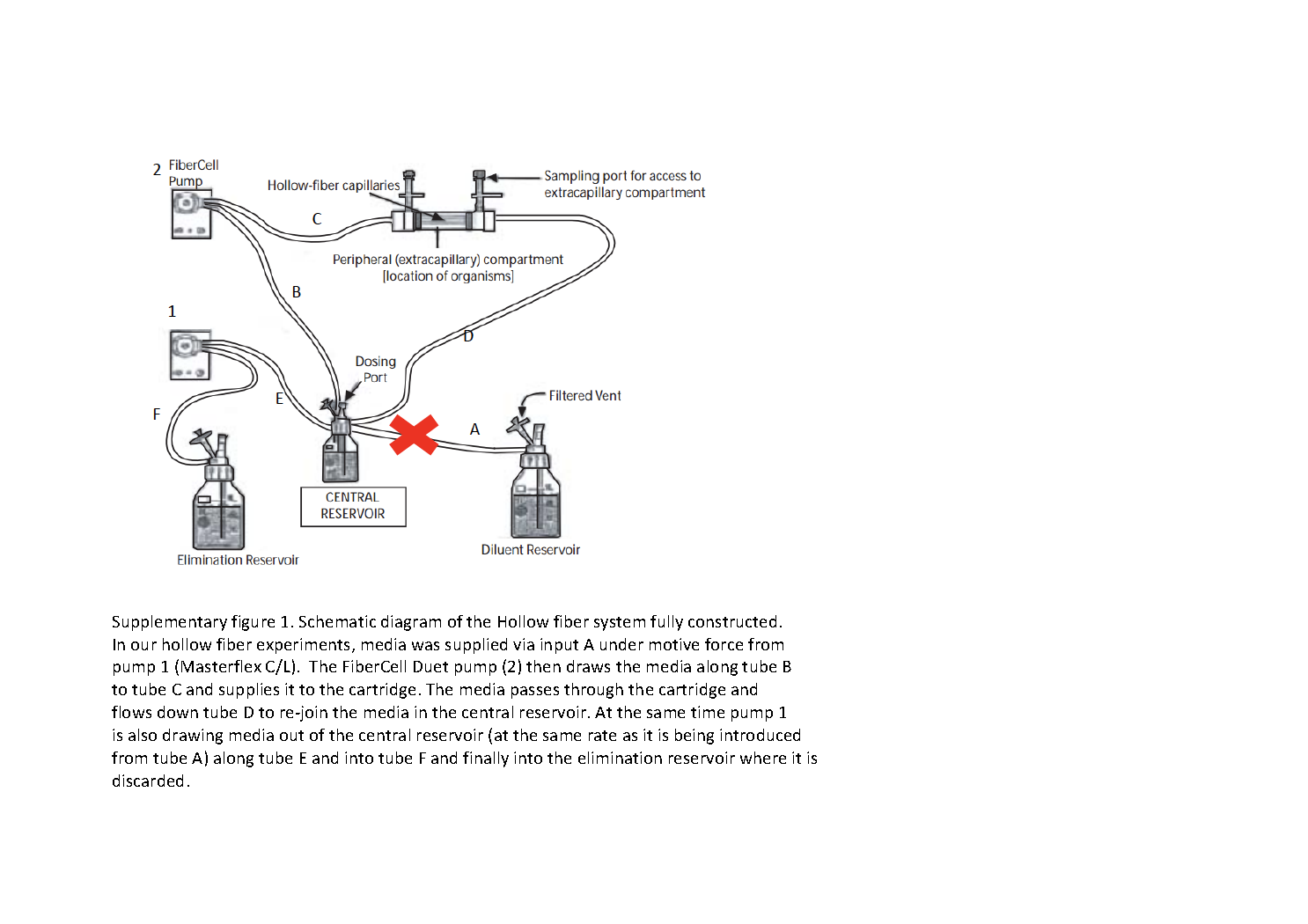

Supplement: Supplementary file 1 [file Image_1.tiff]

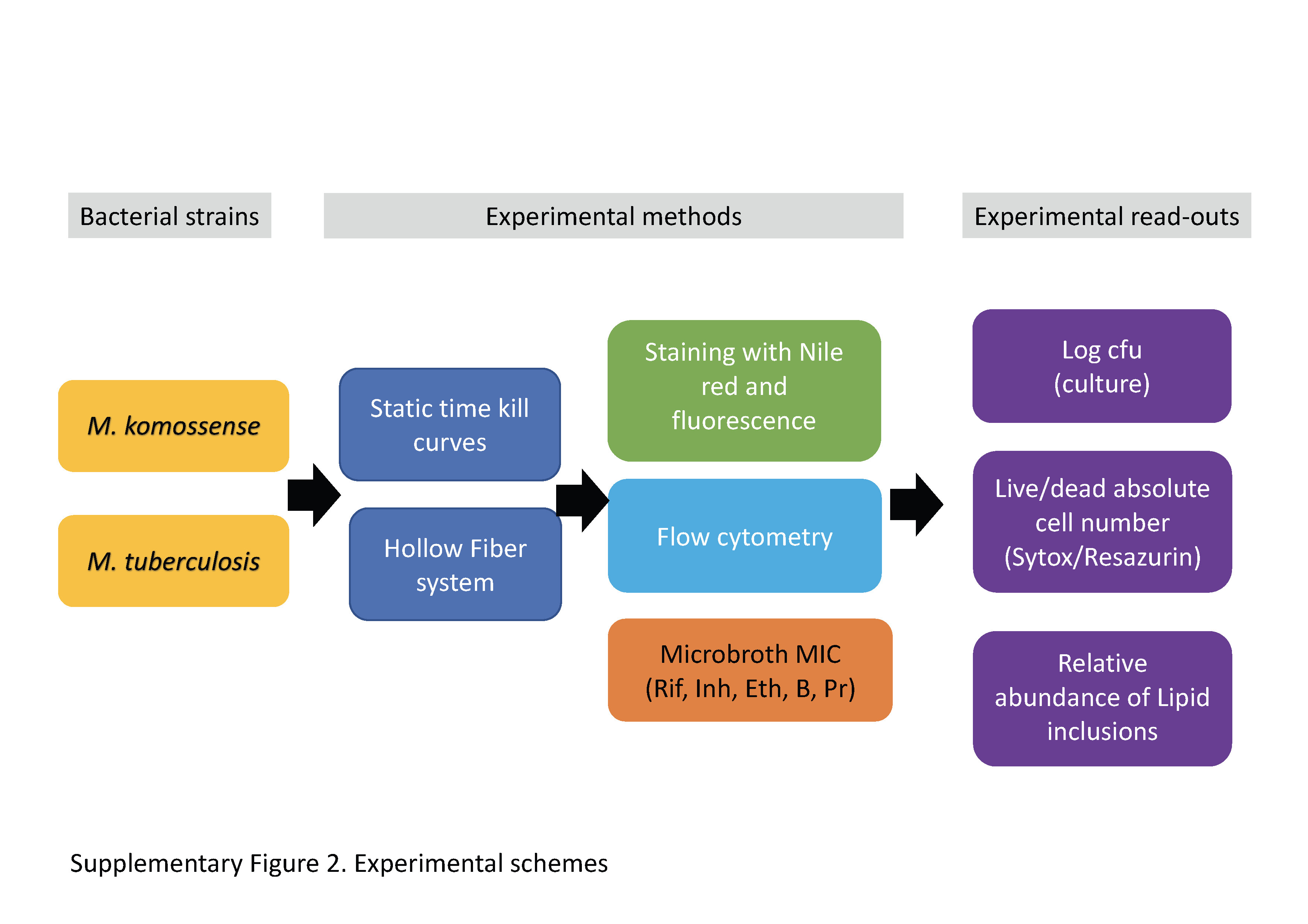

Supplement: Supplementary file 2 [file Image_2.tiff]
